# Five-fold symmetry as indicator of dynamic arrest in metallic glass-forming liquids

**DOI:** 10.1038/ncomms9310

**Published:** 2015-09-21

**Authors:** Y. C. Hu, F. X. Li, M. Z. Li, H. Y. Bai, W. H. Wang

**Affiliations:** 1 Institute of Physics, Chinese Academy of Sciences, Beijing 100190 China; 2 Department of Physics, Beijing Key Laboratory of Opto-electronic Functional Materials and Micro-nano Devices, Renmin University of China, Beijing 100872 China

## Abstract

With sufficient high cooling rates, a variety of liquids, including metallic melts, will cross a glass transition temperature and solidify into glass accompanying a marked increase of the shear viscosity in approximately 17 orders of magnitude. Because of the intricate atomic structure and dynamic behaviours of liquid, it is yet difficult to capture the underlying structural mechanism responsible for the marked slowing down during glass transition, which impedes deep understanding of the formation and nature of glasses. Here, we report that a universal structural indicator, the average degree of five-fold local symmetry, can well describe the slowdown dynamics during glass transition. A straightforward relationship between structural parameter and viscosity (or α-relaxation time) is introduced to connect the dynamic arrest and the underlying structural evolution. This finding would be helpful in understanding the long-standing challenges of glass transition mechanism in the structural perspective.

## Abstract (web summary)

The structural origin of the dynamic slow down during glass transition remains an open question because of the lack of atomic-scale elucidation. Here, Hu *et al.* propose a parameter to link the structural evolution of the average degree of five-fold local symmetry to dynamic arrest in metallic liquids.

## Graphical abstract

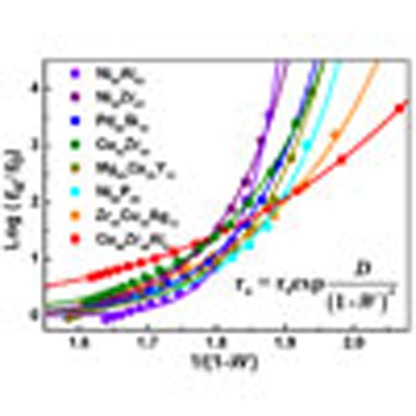

A shape with five-fold rotational symmetry can be mapped onto itself through rotation about a central point by angle of 72° (2*π*/5). About 400 years ago, Kepler had ever found the symmetry of the five Platonic polyhedra in the structure of the solar system, which was associated with the orderly arrangements of plane pentagons^1^. In fact, the five-fold symmetry is ubiquitous in nature and exhibits aesthetic sense, for example, the armour of pineapples, cross-sections of apples, flowers, leaf, starfish and architectures. Many plants display five-fold symmetry to arrange petals to get maximum sunlight without shading each other, showing its significance in natural evolution^2^. In crystallography, the five-fold symmetry once confused people because of its incompatibility with translational periodicity until the discovery of quasicrystals^2^,^3^.

Several decades ago, it was conjectured that liquids may contain many configurations with five-fold symmetry^4^. Recently, by means of advanced instruments, the five-fold symmetry has been experimentally confirmed to exist in liquids^5^,^6^,^7^,^8^,^9^,^10^, colloids^11^,^12^, granular particles^13^, hard-sphere glasses^14^ and metallic glasses (MGs)^15^. Plenty of studies from the perspective of local potential energy minimum and orientational order parameters have been devoted to the effect of the five-fold symmetry on properties, especially in colloidal and granular systems^11^,^13^,^16^,^17^. It is found that because of the incompatibility with translational symmetry, the five-fold symmetry results in severe frustration and hinders crystallization in colloids^12^,^16^. Moreover, the five-fold symmetry is verified to play a crucial role in dynamical arrest in colloidal and granular systems^12^,^13^ and closely correlated with some properties such as fragility and boson peak^16^,^17^. This indicates that the five-fold symmetry may be a good structural parameter for establishing the structure–property relationship.

Unlike colloidal systems, the five-fold symmetry is difficult to be directly observed in MG-forming liquids. However, studies have indicated that the atomic symmetry in MG-forming liquids plays important roles in mechanical properties and glass-forming ability^18^,^19^,^20^,^21^,^22^,^23^,^24^,^26^. On the other hand, plenty of studies via computer simulations have found that icosahedral clusters with high degree of five-fold symmetry play an unique role in dynamics and mechanical properties^7^,^22^,^26^,^27^. Nevertheless, metallic liquids and glasses have diverse atomic clusters. Even for the icosahedral clusters, they are found to be distorted and show diverse configurations in MGs^15^. Therefore, metallic liquids and glasses cannot be modelled by a uniquely prescribed stereochemical structure^26^,^28^,^29^.

For an atomic cluster, the structural configuration can be characterized by the Voronoi tessellation^26^ in terms of Voronoi polyhedron. Each polyhedron mainly contains four types of polygons, that is, triangle, tetragon, pentagon and hexagon. It has been proved that the pentagonal structure has lower potential energy and higher packing density, denoting more stable configurational state^3^,^12^,^13^,^19^,^20^. Furthermore, pentagons representing the five-fold symmetry exhibit totally different temperature-dependent behaviour in glass formation and mechanical response to the deformation in CuZr system^18^,^20^,^24^. It is also found that the plastic events prefer to be initiated in regions with lower degree of five-fold symmetry and propagate towards regions with higher degree of five-fold symmetry^24^, exhibiting the significance of the five-fold symmetry in MGs. Therefore, it is essential to investigate the effect of the five-fold symmetry on the dynamics in MG-forming liquids.

The aim of this paper is to define a structural indicator, five-fold local symmetry, and thereby establish an explicit relationship between structural evolution and dynamic arrest in MG-forming liquids. Owing to the difficulty in experimentally detecting the five-fold symmetry in metallic liquids and glasses, the classical molecular dynamics (MD) simulations with embedded-atom method (EAM) potentials were employed to investigate structural evolution during the dynamic arrest in several typical MG-forming systems. A structural parameter *W* is defined to quantitatively describe the average degree of five-fold symmetry in MG-forming liquids and employed to establish the relationship between structure and dynamics during glass formation. We show that the parameter *W* can depict the marked arrest in MG-forming liquids, and a straightforward relationship between the proposed structural parameter and the drastic dynamic arrest as well as the underlying structural evolution for the metallic liquids is deduced. This quantitative relationship can also reflect the structural heterogeneity basis of the dynamical heterogeneity and thermodynamic characteristics during glass transition. The results might be also suitable for other amorphous systems and helpful in understanding the long-standing challenges of glass transition mechanism in the structural perspective.

## Results

### Definition of the average degree of five-fold local symmetry

Classical MD simulations were performed to generate three-dimensional atomic structures during glass formation (Methods). The local atomic structures were further characterized by employing the Voronoi tessellation^24^ (Methods). Based on the Voronoi analysis, we define the average degree of *κ*-fold local symmetry as 
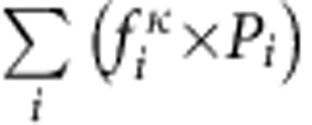
, where *P*_*i*_ is the fraction of polyhedron type *i* and 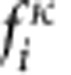 represents the fraction of *κ*-edged polygon in Voronoi polyhedron type *i* and is defined as 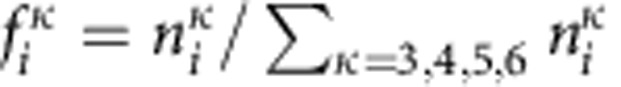 (ref. 29). Here 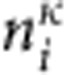 denotes the number of *κ*-edged polygon in Voronoi polyhedron type *i*. It has been confirmed that while 3-, 4- and 6-fold local symmetries are decreasing as temperature decreases, 5-fold local symmetry increases as temperature is approaching *T*_g_ (Supplementary Fig. 1). This is consistent with previous simulation results^20^. It indicates that the five-fold local symmetry may play an essential role in glass formation. Therefore, we define a parameter *W* as the average degree of the five-fold local symmetry:


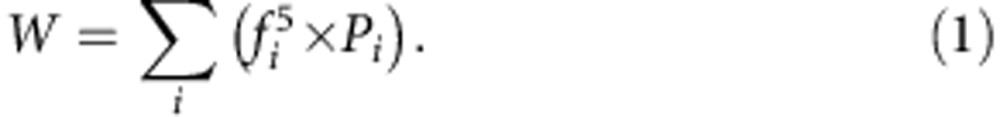


**Figure 1:**
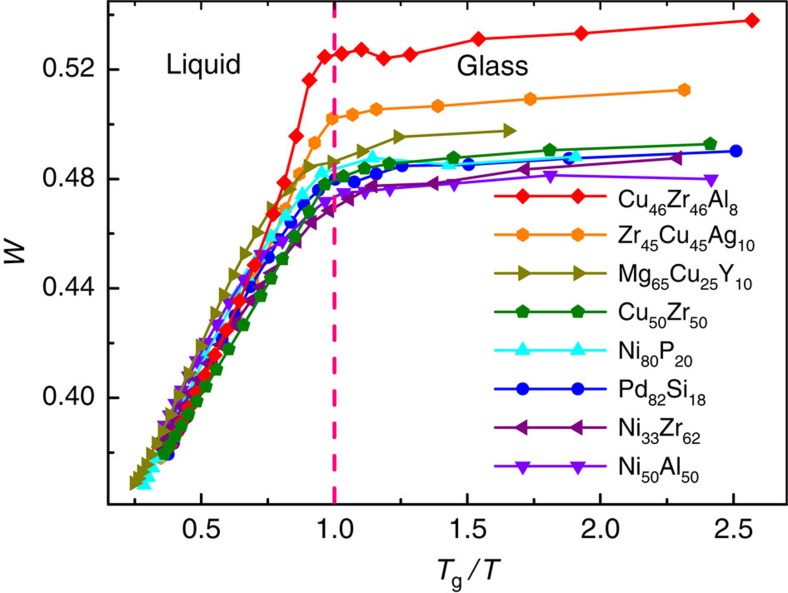
The evolution of five-fold local symmetry during quenching. The temperature dependence of *W* for the simulated systems showing similar trend but different values after glass transition.

To verify the universality of this structural parameter in amorphous state, eight typical MG-forming liquids, including NiP, NiZr, NiAl, PdSi, CuZrAl, ZrCuAg and MgCuY, were investigated (Methods). The total pair correlation functions of the simulated systems at 300 K (Supplementary Fig. 2) are different from each other manifesting different structures, which indicates the diversity of the investigated MG samples^30^. Figure 1 shows the temperature dependence of *W* in the simulated MG-forming liquids. It is clearly seen that *W* in all the simulated systems exhibits similar temperature-dependent behaviour, increasing rapidly above *T*_g_ and reaching constant values below *T*_g_. However, the constant values of *W* below *T*_g_ are different in different metallic liquids, indicating that the degree of the five-fold symmetry is a material-dependent property and could be used to distinguish different MGs^12^. The temperature dependence of *W* represents the structural evolution of MG-forming liquids during glass transition.

### Structure–dynamics relation

**Figure 2:**
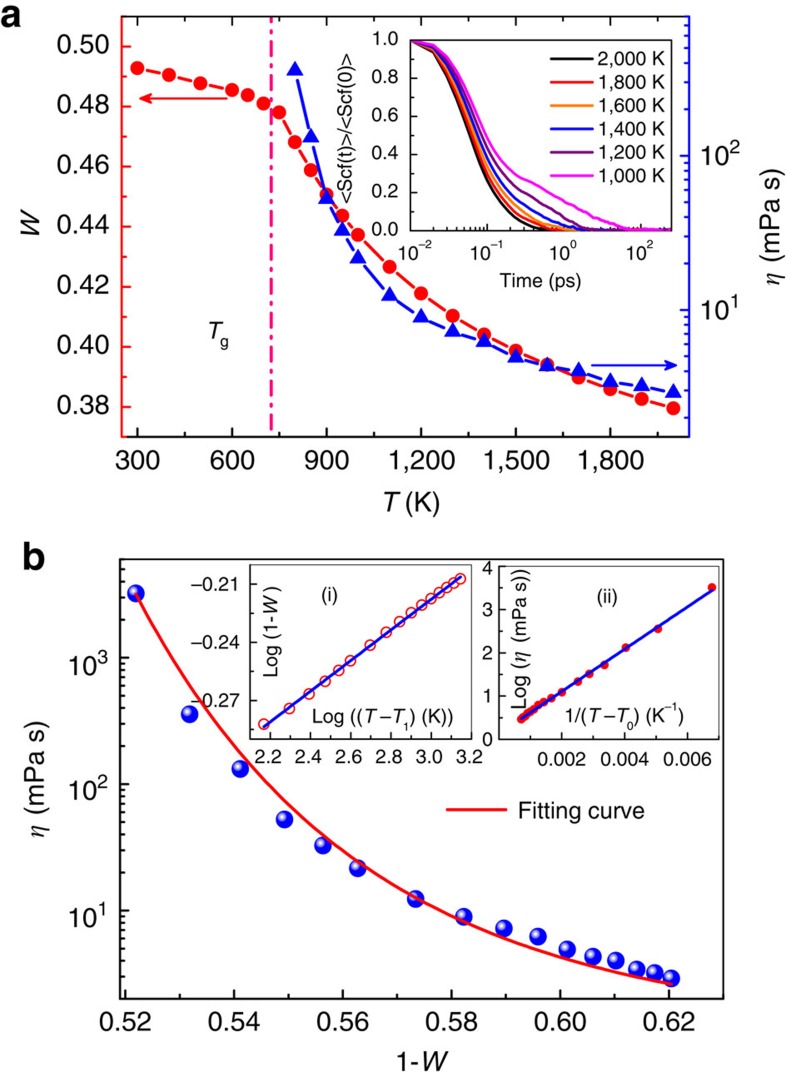
Relationship between viscosity and average five-fold local symmetry. (**a**) The variation of *W* and the shear viscosity with temperature for Cu_50_Zr_50_ metallic liquid. The inset shows the normalized shear auto-correlation function, which was used to calculate the shear viscosity; (**b**) shear viscosity *η* versus *W*. The dotted lines are obtained from simulations and the solid line is the fitting by equation (3); in the insets (i) and (ii), the open and solid circles show the temperature dependence of *W* and *η*, respectively, and the solid lines are fitting of the power-law and VFT functions, respectively.

To clarify the relation between dynamics and structure, the shear viscosity was calculated for the model system of Cu_50_Zr_50_ metallic liquid with the equilibrium MD (Green–Kubo theorem) method based on the shear auto-correlation function^31^ (as shown in the inset of Fig. 2a),


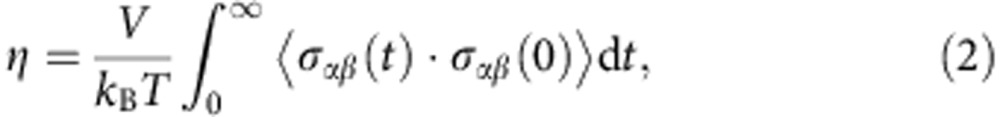


where *η* is the shear viscosity, *V* is the volume, *k*_B_ is the Boltzmann constant and *T* is temperature. 
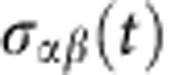
 represents the off-diagonal components of stress tensor at time *t*, and 
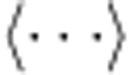
 denotes ensemble average. Figure 2a shows the temperature dependence of shear viscosity above *T*_g_ in Cu_50_Zr_50_ metallic liquid. The temperature dependence of *W* was also presented for comparison. As temperature approaches *T*_g_, shear viscosity drastically increases, indicating dynamical slowdown during glass formation. As shown in Fig. 2a, it is obvious that *η* and *W* show a similar trend as temperature is approaching *T*_g_, indicating that there could exist a link between the underlying elementary structural evolution and the change in viscosity. Figure 2b shows the variation of viscosity with *W* in Cu_50_Zr_50_ metallic liquid. As *W* increases, *η* is increasing. To establish a quantitative link between *η* and *W*, a power law of 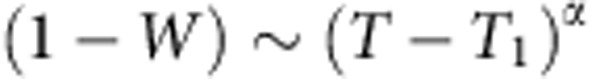 and the Vogel–Fulcher–Tammann (VFT) equation 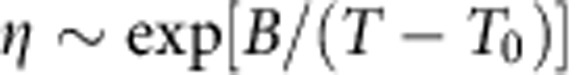 were employed to fit *W* and *η* as a function of temperature, respectively^32^. As illustrated in the inset in Fig. 2b, the simulated data are fitted very well by the power-law function and VFT equation, respectively, and the statistical correlation parameter *R*^*2*^ is better than 0.99. As a result, *T*_1_ is approximately equal to the ideal glass transition temperature *T*_0_ (*T*_1_≈*T*_0_≈0.85*T*_g_ (ref. 33)). By substituting *T*_0_ in VFT equation with *T*_1_, temperature can be eliminated and a direct relationship between *W* and *η* is deduced:


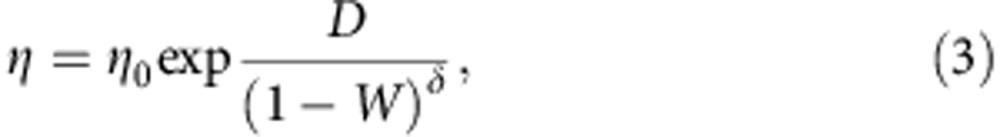


where *η*_0_ is the viscosity at infinite liquidus temperature, *D* and *δ* are fitting parameters. To verify the validity of equation (3), the viscosity against (1-*W*) shown in Fig. 2b was fitted with equation (3). Apparently equation (3) describes the behaviour of viscosity as a function of *W* very well. We note that the fitting parameter *δ*≈12.30 for Cu_50_Zr_50_ metallic liquid, much larger than 1, indicating that slight change in structure will lead to marked change in viscosity. Therefore, *δ* reflects the sensitivity of the viscosity change to the local structure change. The larger the *δ* value is, the more drastically the viscosity changes with *W.* The evolution of structure is the intrinsic reason of the dynamic slow down, and the ‘hidden' structure changes responsible for the extremely large variation in dynamics of supercooled liquids can be distinctly described by the structural parameter *W*.

**Figure 3:**
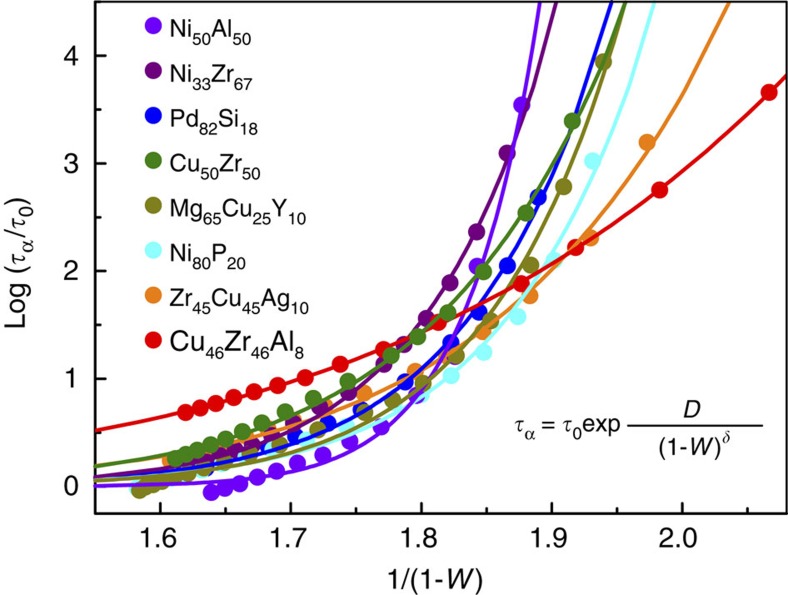
Relation between structure parameter *W* and α-relaxation time *τ*_α_. The dotted and solid curves are simulation data and the fittings with equation (5), respectively.

Since a simple relation between shear viscosity *η* and α-relaxation time (*τ*_α_) exists, equation (3) should also be applicable to *τ*_α_. Instead of shear viscosity, in the following we will focus on α-relaxation time *τ*_α_, as it is more easily computed. A common measurement of *τ*_α_ is the time when the self-intermediate scattering function *F*_s_(*q, t*) decays to *e*^−1^ of its initial value:


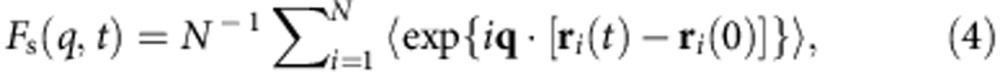


where *N* is the atom number, **r**_***i***_ is the atomic position of atom *i* and **q** is the wave vector fixed at *q*_max_*=|***q***|* corresponding to the first peak position of structure factor^22^,^34^. Supplementary Fig. 3 shows the typical behaviour of *F*_s_(*q,t*) as a function of *t* at various temperatures in Cu_46_Zr_46_Al_8_ metallic liquid, from which the α-relaxation times at different temperatures can be extracted. The temperature dependence of *τ*_α_ for various MG-forming liquids shown in Supplementary Fig. 4 can be well fitted by the VFT equation: 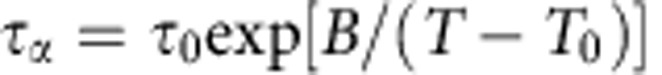, where *T*_0_ is approximately equal to *T*_1_ in the power law of 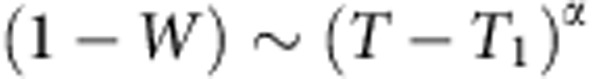. Therefore, equation (3) can be indeed applied to describe the relationship between *τ*_*α*_ and *W* as,


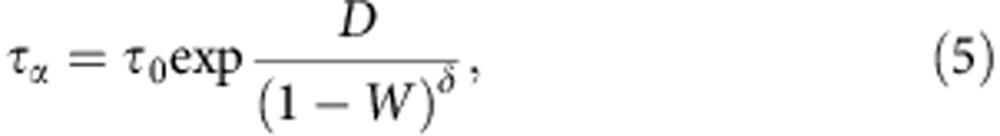


where *τ*_0_ is the relaxation time at infinite liquidus temperature, and *δ* and *D* are fitting parameters. Figure 3 illustrates the α-relaxation time *τ*_α_ as a function of *W* and the fittings of equation (5) for various MG-forming liquids (*R*^2^≥0.99 for all the fittings). Remarkably, equation (5) can well describe the relationship between structural relaxation time and the five-fold local symmetry in MG-forming liquids. In addition, *δ* is fitted to be about 6.78, 11.52, 13.58, 17.65, 17.95, 18.92, 18.94, and 32.74 for Cu_46_Zr_46_Al_8_, Zr_45_Cu_45_Ag_10_, Cu_50_Zr_50_, Ni_80_P_20_, Pd_82_Si_18_, Mg_65_Cu_25_Y_10_, Ni_33_Zr_67_ and Ni_50_Al_50_ MG-forming liquids, respectively. Clearly, *δ* is quite different for different systems, whereas the fitting parameter *D* is found to be similar (∼10^−5^) in different systems. As mentioned above, *δ* reflects the sensitivity of the viscosity or α-relaxation time to structure change. Therefore, the effect of the structure change on the relaxation dynamics is significantly different in different MG-forming liquids. Our results show that there exists a universal underlying structural evolution in MG-forming liquids, which is responsible for the marked dynamic slowdown. The results are also in agreement with the observation of locally favoured structure in colloidal gels during gelation^12^ and granular systems^13^, and medium-range crystalline order in granular liquids during liquid–glass transition^35^.

### Atomic mobility

**Figure 4:**
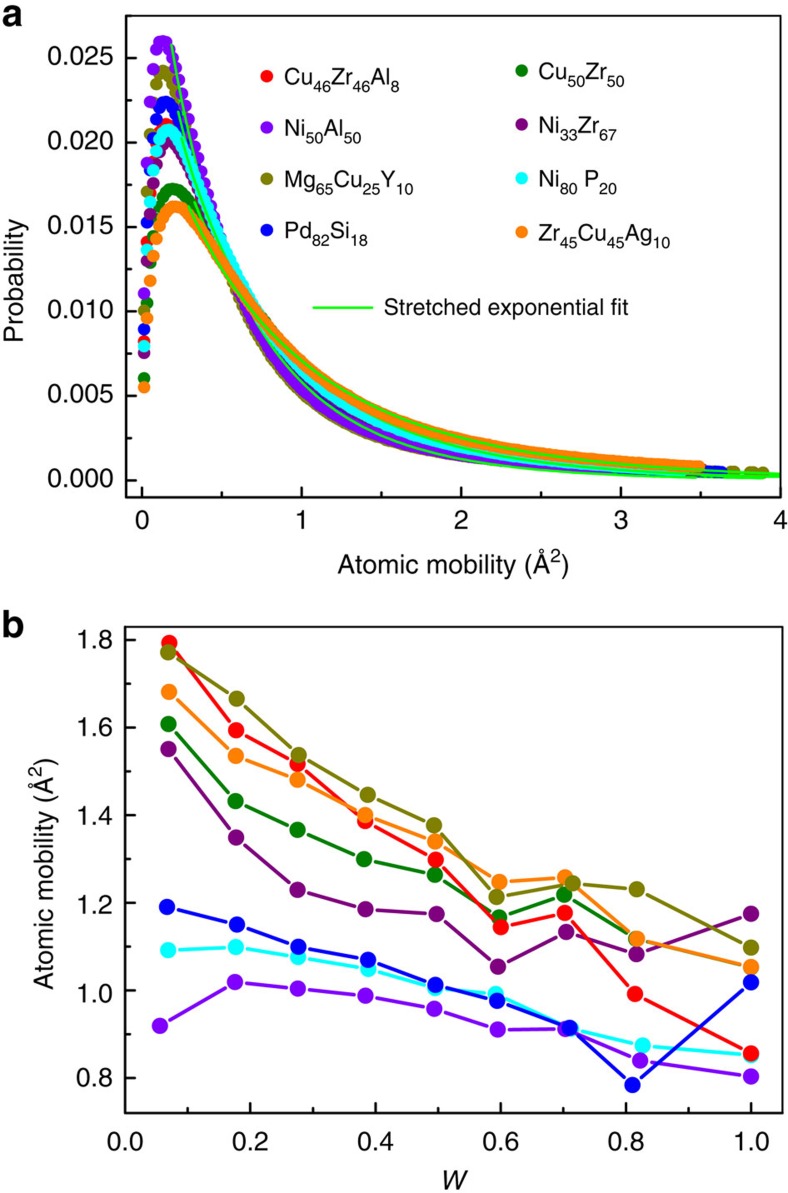
Correlation between structure parameter *W* and atomic mobility. (**a**) Distribution of atomic mobility at *T*=1.2*T*_g_ in different systems. There is a long tail in the distribution for all the simulated systems, which can be fitted by a stretched exponential function 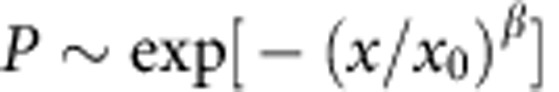 with 0<*β*<1 (green solid lines), indicating heterogeneous dynamics. (**b**) The dependence of atomic mobility on *W* in various simulated systems (the same colour in **a**,**b** represents the same system).

The atomic mobility can reflect the effect of local structure on the dynamical behaviour of atoms. The non-Gaussian parameter 
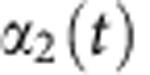
 (ref. 34), 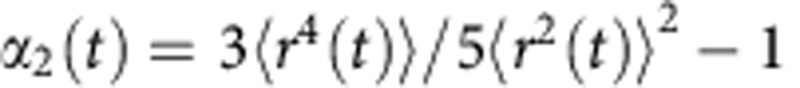, is commonly believed to reflect dynamical heterogeneity in supercooled liquids. The peak time t* in 
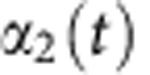
 represents the timescale at which the distribution of atomic motion is the most heterogeneous^22^. The atomic mobility of atom *i* can then be evaluated in the time interval of *t*=t* according to 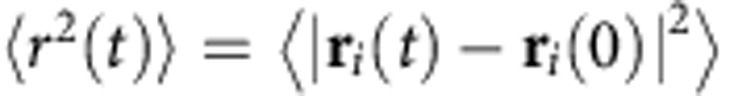 for various MG-forming liquids. Figure 4a shows the distribution of atomic mobility in the investigated systems at *T*=1.2*T*_g_. It is clear that the distributions of atomic mobility with very long tails significantly deviate from Gaussian distribution, indicating the inhomogeneous dynamics in the supercooled MG-forming liquids, which is in agreement with previous results^22^,^36^,^37^,^38^. Furthermore, the long tails in the distribution can be well fitted with a stretched exponential function 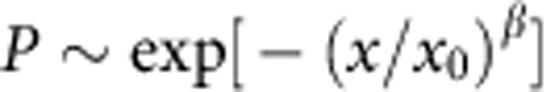, as shown in Fig. 4a. The parameter *β* was fitted to be about 0.77, 0.86, 0.81, 0.84, 0.82, 0.75, 0.83 and 0.83 for Cu_46_Zr_46_Al_8_, Zr_45_Cu_45_Ag_10_, Cu_50_Zr_50_, Ni_80_P_20_, Pd_82_Si_18_, Mg_65_Cu_25_Y_10_, Ni_33_Zr_67_ and Ni_50_Al_50_, respectively. These values are all smaller than 1, further indicating the heterogeneous dynamics in all the supercooled MG-forming liquids^37^. In the following, we will establish a link between atomic mobility and the five-fold local symmetry.

Figure 4b shows the correlation between five-fold local symmetry and atomic mobility for these metallic liquids at *T*=1.2*T*_g_. It is shown that the atomic mobility decreases with increasing *W*. This behaviour also holds well at other temperatures and becomes more remarkable with decreasing temperature (Supplementary Fig. 5). The more the five-fold symmetry in atomic packing around atoms, the more immobile the atoms are. This clearly demonstrates that atomic mobility in MG-forming liquids directly correlates to the five-fold local symmetry, and the degree of the five-fold symmetry in local atomic packing does impact the mobility of the involved atoms.

### Spatial structure correlation

**Figure 5:**
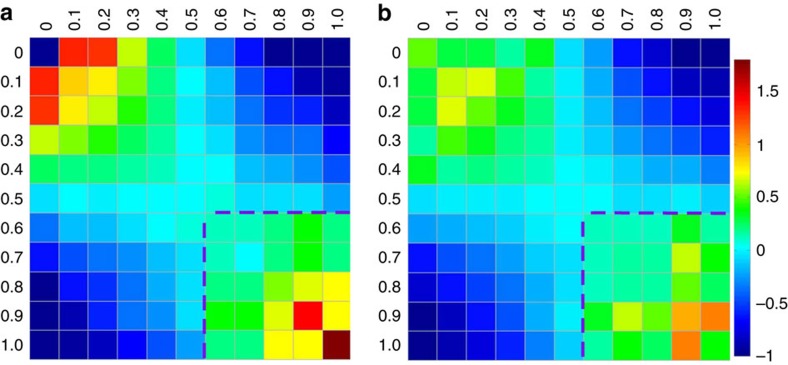
Correlation matrix of atoms with diverse *W*. Correlation matrices of *C*_*ij*_ between atoms classified into diverse *W* in (**a**) Cu_46_Zr_46_Al_8_ and (**b**) Mg_65_Cu_25_Y_10_ at *T*=1.2*T*_g_ (the behaviour of *C*_*ij*_ is similar at different temperatures).

To get deep insight into the correlation between *W* and dynamics, we analysed the spatial structures related to the different degree of five-fold local symmetry. Here a nearest-neighbour correlation index *C*_*ij*_ defined as 
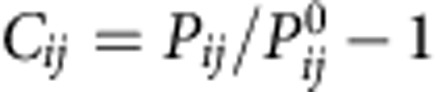
 was adopted^39^, which reflects the spatial correlation between central atoms of polyhedra *i* and *j*, where *P*_*ij*_ and 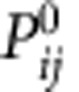 represent the probability of polyhedra types *i* and *j* being the nearest neighbours in real structure model and in the case of the distributions of indices are spatially random, respectively. Positive values of *C*_*ij*_ indicate strong correlation between polyhedra *i* and *j* and vice versa. Figure 5 shows the correlation matrix of *C*_*ij*_ between atoms with classified *W* values at 1.2*T*_g_ for Cu_46_Zr_46_Al_8_ and Mg_65_Cu_25_Y_10_ MG-forming liquids, respectively (see Supplementary Fig. 6 for other systems). It is clearly seen that the correlation matrix of *C*_*ij*_ exhibits similar patterns in various MG-forming liquids, although the structural motifs in these systems are different^10^. The correlation patterns are also similar at different temperatures (not shown). This indicates that the structural parameter *W* is generic in describing the structure properties and spatial structure correlations in MG-forming liquids. The values of *C*_*ij*_ in the upper-left and bottom-right corners in the correlation maps are larger than 0, whereas those in the upper-right and bottom-left corners are smaller than 0. This implies that the atoms with *f*^5^≥0.6 or *f*^5^≤0.4 tend to form clusters, whereas the atoms with *f*^5^≥0.6 and *f*^5^≤0.4 tend to avoid each other.

**Figure 6:**
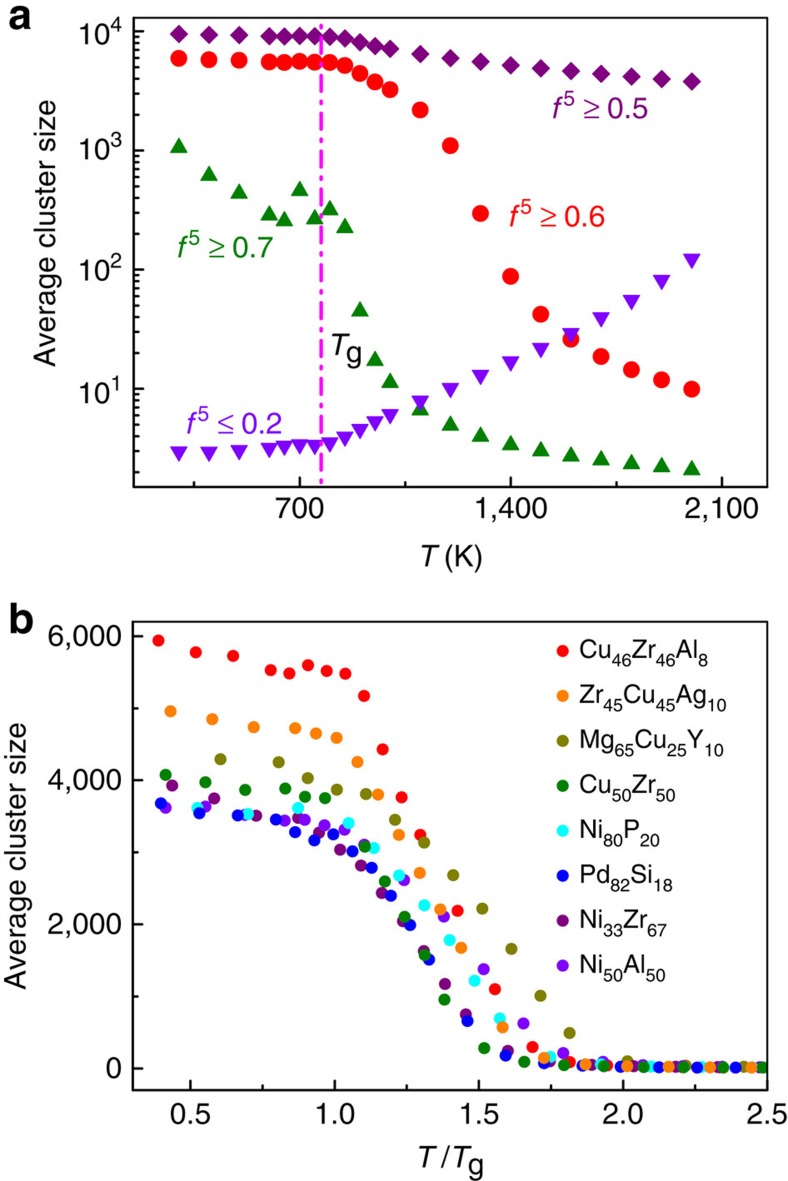
The temperature dependence of the average cluster size. (**a**) The average cluster size formed by atoms with different thresholds of *W* in Cu_46_Zr_46_Al_8_ metallic glass-forming liquid, showing only the selection of 0.6 is reasonable. The inverse tendency of average cluster size for *f*^5^≥0.6 and *f*^5^≤0.2 indicates the existing competition between the incompatible symmetry during glass transition. (**b**) The evolution of the average cluster size with decreasing temperature in different systems. Temperature was scaled by *T*_g_. Above *T*_g_, the average cluster size increases remarkably while levels off after glass transition corresponding to the frozen structure in the glassy state.

To unravel the spatial structure correlation, cluster analysis was conducted to investigate the cluster size evolution with temperature decreasing for different threshold of five-fold local symmetry^40^. A cluster can be defined if atoms with the same threshold are nearest neighbours, and the cluster size is defined as the number of contained atoms. Thus, the average cluster size was calculated according to 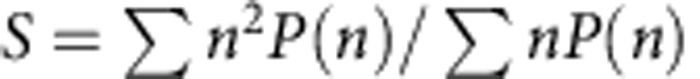, where *n* is the individual cluster size and *P*(*n*) is its probability^40^. The thresholds of *f*^5^≥0.5, 0.6 and 0.7 were chosen for cluster analysis. The threshold of *f*^5^≤0.2 was also tested for a comparison. As shown in Fig. 6a, the average cluster size is increasing for the threshold of *f*^5^≥0.5, 0.6 and 0.7, respectively, during quenching, whereas the average cluster size of *f*^5^≤0.2 is decreasing. This indicates that the population of the Voronoi clusters with higher degree of five-fold local symmetry is increasing with decreasing temperature and form larger and larger clusters, whereas the population of the Voronoi clusters with lower degree of five-fold local symmetry decreases.

It is clearly shown that the average cluster size of *f*^5^≥0.5 is too large at high temperature range, containing more than 1,000 atoms, which is unphysical, whereas the average cluster size of *f*^5^≥0.7 is relatively small below *T*_g_, which is unphysical either, because it increases too slowly as temperature decreases and still varies when temperature is below *T*_g_ (see Fig. 6a). For *f*^5^≥0.6, however, the average cluster size exhibits reasonable temperature-dependent behaviour: relatively small (∼10 atoms) at high temperature, increasing drastically as temperature approaches *T*_g_ and reaching a constant value below *T*_g_. The rationality of the choice of *f*^5^≥0.6 was also confirmed by the nearest-neighbour correlation index as illustrated in Fig. 5 and Supplementary Fig. 6. To further rationalize the choice of *f*^5^≥0.6, we also investigated the changes of the structural relaxation time with average cluster size at different temperatures. Supplementary Fig. 7 shows the α-relaxation time as a function of the cluster size for *f*^5^≥0.5, 0.6 and 0.7 in Cu_46_Zr_46_Al_8_ MG-forming liquids, respectively, which clearly demonstrates that the choice of 0.6 is more reasonable, although it is not absolutely precise. Therefore, the threshold of *f*^5^≥0.6 was chosen in the cluster analysis.

Figure 6b shows the temperature dependence of the average cluster size with *f*^5^≥0.6 for various metallic glass-forming liquids, and similar behaviours were observed. In high temperature range, the average cluster size is quite small and similar in different systems, but increases drastically as temperature decreases, and finally reaches a constant value as temperature is below *T*_g_. We also examined the growth of the largest cluster during glass formation. Supplementary Fig. 8 shows the snapshots of the largest cluster formed by atoms with *f*^5^≥0.6 in Cu_46_Zr_46_Al_8_ MG-forming liquid during quenching. At high temperature such as 2.5*T*_g_, the largest cluster is very small, containing only about 20 atoms. As temperature decreases to 2.0*T*_g_, the size of the largest cluster increases to 100–200 atoms. As temperature decreases further to 1.5*T*_g_, the largest cluster forms a network-like structure and almost percolates as shown in Supplementary Fig. 8. As temperature is below *T*_g_, the largest cluster almost fills up the whole space as shown in Supplementary Fig. 8. Similar evolution behaviour of the largest cluster with decreasing temperature is also observed in other MG-forming liquids (not shown). Therefore, the percolation of the largest cluster formed by the atoms with *f*^5^≥0.6 during glass transition^12^ stabilizes the whole system and drastically slows down the dynamics because of their low atomic mobility (Supplementary Fig. 9).

### Thermodynamics

**Figure 7:**
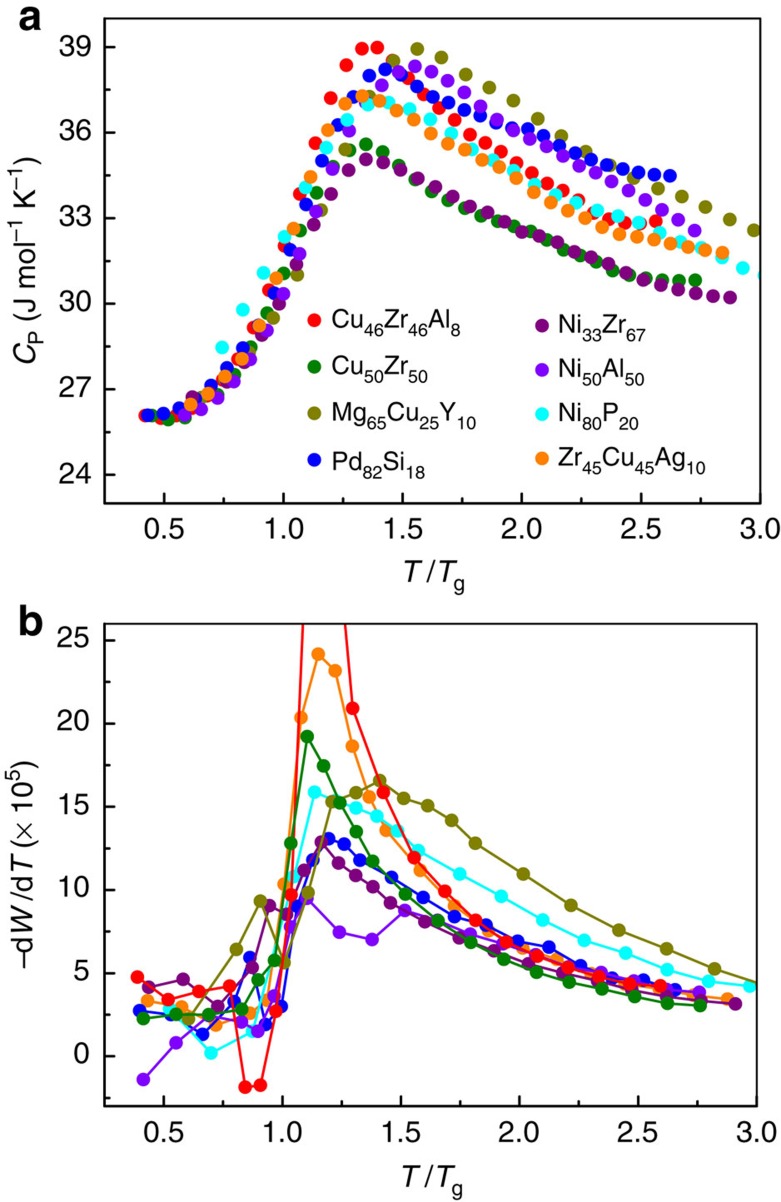
Correlation between specific heat *C*_P_ and *W*. (**a**) The temperature dependence of the specific heat *C*_P_ for various systems. (**b**) The structural change rate during quenching reflected by d*W*/d*T* shows a jump during glass transition. The coincidence of the excess specific heat and the jump in d*W*/d*T* illustrates the structural basis of the thermodynamics (the same colour in **a**,**b** represents the same system).

It is commonly believed that vitrification is a result of extreme difficulty in crystal nucleation and growth, during which thermodynamics is also crucial. According to Adam-Gibbs theory^41^, the dynamics correlates strongly with configurational entropy, which is controlled by structural evolution. To unravel the connection among dynamics, thermodynamics and structure, we investigated the correlation between five-fold local symmetry and specific heat. Here the isobaric-specific heat *C*_P_ was calculated in terms of its definition, 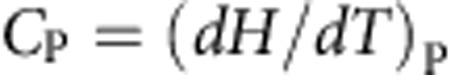, where *H* is the enthalpy^42^,^43^. Figure 7a shows the typical temperature dependence of *C*_P_ during glass transition. Here temperature is scaled by *T*_g_. During quenching *C*_P_ increases and reaches a maximum, then decreases. An excess specific heat was observed in Fig. 7a. If one takes a derivative of *W*, d*W*/d*T*, a similar jump behaviour is also observed in the change rate of the *W* parameter, as shown in Fig. 7b, indicating that there exists a correlation between the thermodynamics and the structure evolution during the glass transition.

## Discussion

When metallic melts are cooled down from high temperatures, two processes of crystallization and vitrification are competing. In crystallization, the crystalline structures are formed without five-fold symmetry, whereas in vitrification crystallization is suppressed and glassy states are obtained with local structures containing both icosahedral-like (representative of five-fold symmetry) and fcc-like structural features^15^,^44^. Therefore, the vitrification process can be regarded as the competition between five-fold symmetry and crystal symmetry, and the five-fold symmetry increases in vitrification (Supplementary Fig. 1 and Fig. 6)^44^, reflecting the underlying structural evolution for the dynamical arrest in MG-forming systems^16^,^26^. Although the distinct structures and properties are observed in the various simulated systems, equations (3) and (5) reveal proper and universal structure–dynamics relation in MG-forming liquids, analogous to the structural mechanism for dynamic arrest in colloidal and granular systems^12^,^13^. It confirms that the five-fold symmetry *W* can characterize the underlying structural evolution and the structural mechanism of glass transition, and shows that vitrification is the result of the evolution of the incompatible rotational symmetry competing with long-range translational symmetry for forming crystals^44^.

It is known that clusters with large *W* values such as icosahedra have large packing density comparable to that of the face-centred cubic and hexagonal close-packed, and are lack of translational symmetry, which could result in severe frustration and difficulty to grow compared with their crystalline counterparts^16^,^19^,^20^,^22^,^26^,^44^. In addition, local structure with higher *W* has lower potential energy and configurational entropy resulting in more stable state^12^,^20^. Hence, from the potential energy landscape perspective^45^,^46^, local structure with higher *W* locates in a deeper local minimum with higher energy barrier indicating lower atomic mobility^12^ as shown in Fig. 4b. Because of the enhanced *W* during quenching (Fig. 1), dynamics will slow down drastically. According to Adam-Gibbs theory^41^, the spatial structural correlation increases with decreasing temperature, and results in the heterogeneous dynamics as revealed in Fig. 4a. Our results show that the average cluster size with the threshold of *f*^5^≥0.6 increases during quenching and levels off after glass transition. Percolation may occur approaching *T*_g_ based on the observation of the network-like structure of the largest cluster formed by atoms with high degree of the five-fold symmetry, which was confirmed experimentally in other amorphous systems during glass transition^12^,^13^.

The specific heat jump during glass transition is surprisingly coincident with the jump of the derivative of *W* (d*W*/d*T*), suggesting that the excess specific heat is intimately related to the five-fold symmetry evolution, which controls the configurational freedom. Such a relation is quite reasonable based on the assumption that the entropy change near glass transition is mainly due to the configuration change, which is a basic hypothesis in many glass transition theories such as Adam-Gibbs model^41^. If the derivative of entropy to temperature in 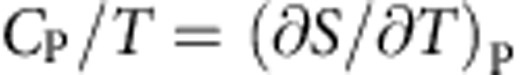 is just the configurational part, 
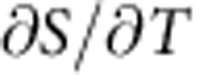
 is controlled by the change rate of structure reflected by d*W*/d*T*, and it is a natural deduction that the specific heat jump should agree well with the slope change of *W*. Based on the Adam-Gibbs model: 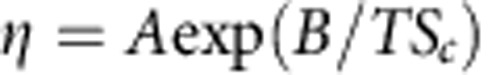
41, the increase of *W* with decreasing temperature may lead to the reduction of configurational entropy and ultimately induce marked dynamic slowdown. The relationship among structure, dynamics and thermodynamics is then correlated with a quantitative structural parameter of the five-fold local symmetry.

It is interesting to note that if *W* in equation (5) goes to zero, that is, *W*→0, the crystalline structure is obtained, since the five-fold symmetry disappears and the corresponding Voronoi polyhedra of crystal-like clusters are <0,12,0,0>, <0,6,0,8>, <0,6,0,2> and so on^42^. For *W*→1, *τ*_α_→∞, which is equivalent to the situation that *T* is approaching *T*_0_, indicating the formation of ideal glass^33^. In the case of *W*→1, the system would possess the highest packing density and the most stable state, corresponding to the deepest potential energy minimum in the potential energy landscape, which is comparable to that of crystals^12^,^45^,^46^. However, full icosahedra with ideal five-fold symmetry (*W*=1) cannot fill the entire three-dimensional space and the multicomponent essence of MGs (frustration) impedes the formation of full icosahedra^26^. Consequently, this implies that ideal glass could have quasicrystal-like structure with a specific fractal nature^30^.

According to equations (3) and (5), given the same change of *W*, the higher *δ* value means the more drastic change of the viscosity or α-relaxation time, which reflects the sensitivity of the dynamics variation to the internal structure change in MG-forming liquids. This is analogous to the dynamic fragility defined as 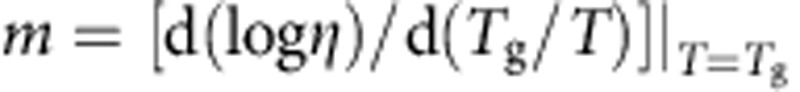 and describes the sensitivity of the dynamics change to the temperature as temperature is approaching *T*_g_ (refs 47, 48). Therefore, *δ* in equations (3) and (5) can be regarded as ‘structural fragility'. Moreover, the dynamic heterogeneity can also be interpreted by the heterogeneous spatial distribution of microstructure as shown in Supplementary Fig. 10, establishing the structural basis of the heterogeneous dynamics. Consequently, structure–dynamics relationship is helpful for understanding the structural heterogeneity basis of dynamic heterogeneity.

In our previous work^49^, a relation, 
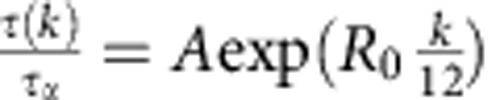
, between the connectivity of icosahedral clusters *k* and the corresponding relaxation time of the related local structures *τ*(*k*) was derived. Here *A* and *R*_0_ are free parameters, *τ*_α_ is the relaxation time of the system. Both *τ*(*k*) and *k* are local quantities. This relationship provides evidence of dynamical heterogeneity and its correlation with the icosahedral medium-range structures in supercooled CuZr glass-forming liquids. However, equation (3) or equation (5) establishes a relation between the overall dynamical properties such as the viscosity or relaxation times and a structural parameter, five-fold local symmetry. Although temperature does not explicitly show up in these equations, both relaxation times or viscosity and *W* change with temperature. Therefore, it can describe the relationship between relaxation time or viscosity and *W* at different temperatures. Therefore, equation (3) or equation (5) is totally different from that in ref. 49 On the other hand, the relation derived in ref. 49 can be applied to quantify the local dynamics of atomic structures formed by atoms with some specific *f*^5^ values. For instance, atoms with *f*^5^≥0.6 tend to form clusters as illustrated in Fig. 5, containing various connectivity degrees, so that the relaxation times of atoms with *f*^5^≥0.6 and different connectivity can be evaluated based on the relation in ref. 49, which may provide more information of the correlation between dynamics and medium-range structures formed by atoms with *f*^5^≥0.6. Note that although *W* values are similar (Fig. 1), the MRO formed by the atoms with larger five-fold local symmetry may be quite different (Fig. 6b), which significantly affects the dynamics and relaxation in MG-forming liquids, according to the relation in ref. 49. In addition, this has also been incorporated into equation (3) or equation (5) through the exponent parameter *δ*, which reflects how sensitively the dynamics varies with structure changes in MG-forming liquids.

We also checked the relation between the fraction of the icosahedral clusters and the relaxation time during glass formation, and fitted the data in terms of equation (5) by substituting *W* with the fraction of the icosahedral clusters. It is found that icosahedral clusters do not work for the fitting. Furthermore, the fitting of icosahedral clusters generates unphysical values of *τ*_0_ (∼10^0^ fs), which are too small, whereas *W* parameter does obtain physical values of *τ*_0_ (∼10^2^ fs), comparable to the time scale of the vibration in solids. This also demonstrates that the structure parameter *W* is generic in describing the structure–property relationship in MG-forming liquids.

In summary, a universal structural parameter *W*, the average degree of five-fold local symmetry is proposed to characterize the underlying structural evolution during glass transition in MG-forming liquids. A simple and straightforward relation between structure and dynamics,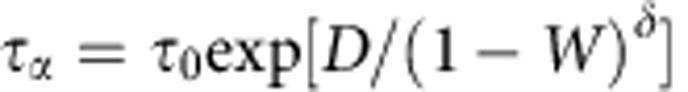, is established implying the structural heterogeneity basis of heterogeneous dynamics. The results confirm that there indeed exists structural characteristic, which is responsible for the markedly dynamical slowdown during glass transition from aspects of atomic mobility, spatial structural correlation and thermodynamics. The results could shed light on the structural mechanism for dynamic arrest in MG-forming liquids and will be helpful in understanding of glassy nature.

## Methods

### MD simulations

In our studies, MD simulations were performed for eight model systems of MG-forming liquids. The EAM potentials were used to describe the interatomic interactions^43^,^50^,^51^,^52^,^53^,^54^,^55^,^56^. All of the simulations were performed using the code LAMMPS and periodic boundary conditions were applied in three dimensions. For each model, the initial configuration containing 16,000 atoms was equilibrated at 2,000 K for 1.5 ns followed by rapid quenching (10^12^ K s^−1^) to 300 K in *NPT* (constant number, constant pressure and constant temperature) ensemble. During cooling, the cell size was adjusted to give a zero pressure and the structure configurations at different temperatures were collected. After adequate relaxation at each temperature of interest, the ensemble was switched to *NVT* (constant number, constant volume and constant temperature) ensemble and each model system was relaxed for 1 ns and 1,000 atomic configurations were collected for structure and dynamics analysis. In the simulations, the time step used to integrate the equations of motion is chosen as 1 fs and the temperature was controlled using the Nose–Hoover thermostat.

### Voronoi tessellation

Voronoi tessellation divides space into close-packed polyhedral around atoms by constructing bisecting planes along the lines joining the central atom and all its neighbours^57^. The Voronoi index 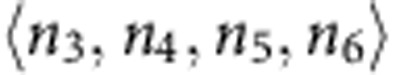 is used to characterize the geometry feature of atomic clusters, where *n*_*i*_ (*i*=3,4,5,6) denotes the number of *i*-edged faces of a Voronoi polyhedron. In our analysis, a cutoff distance of 5 Å was chosen so that the Voronoi index distribution was converged.

## Additional information

**How to cite this article:** Hu, Y. C. *et al.* Five-fold symmetry as indicator of dynamic arrest in metallic glass-forming liquids. *Nat. Commun.* 6:8310 doi: 10.1038/ncomms9310 (2015).

## Supplementary Material

Supplementary Information Supplementary Figures 1-10
