# Supplementary material for: Five-fold symmetry as indicator of dynamic arrest in metallic glass-forming liquids
Source: Nat Commun. 2015 Sep 21;6:8310. doi: 10.1038/ncomms9310 (PMC4595736; doi:10.1038/ncomms9310)
Supplement: Supplementary Information — Supplementary Figures 1-10 [file ncomms9310-s1.pdf]

Supplementary Figures

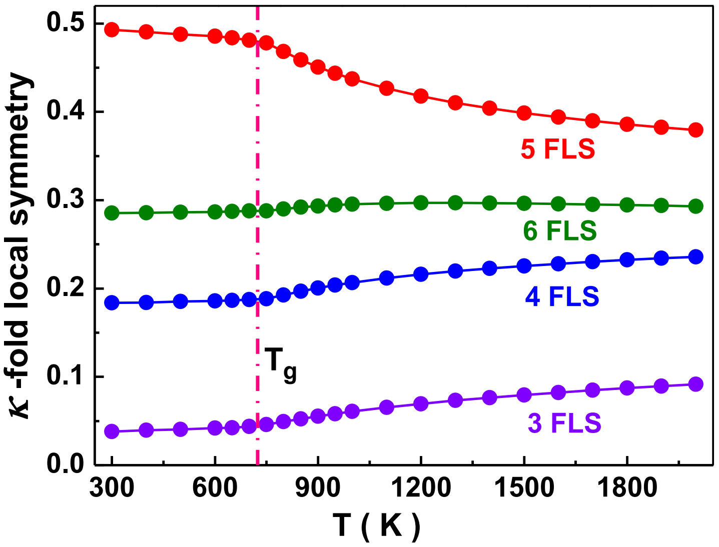

Supplementary Figure 1. The evolution of the averaged κ-fold local symmetry (κFLS, f^{κ}) during quenching in $Cu_{50}Zr_{50}$ metallic glass-forming liquid. While 3-, 4-, and 6-fold local symmetry decreases as temperature is approaching T_g, five-fold local symmetry increases, indicating the critical role of the five-fold local symmetry played in the slowing down dynamics.

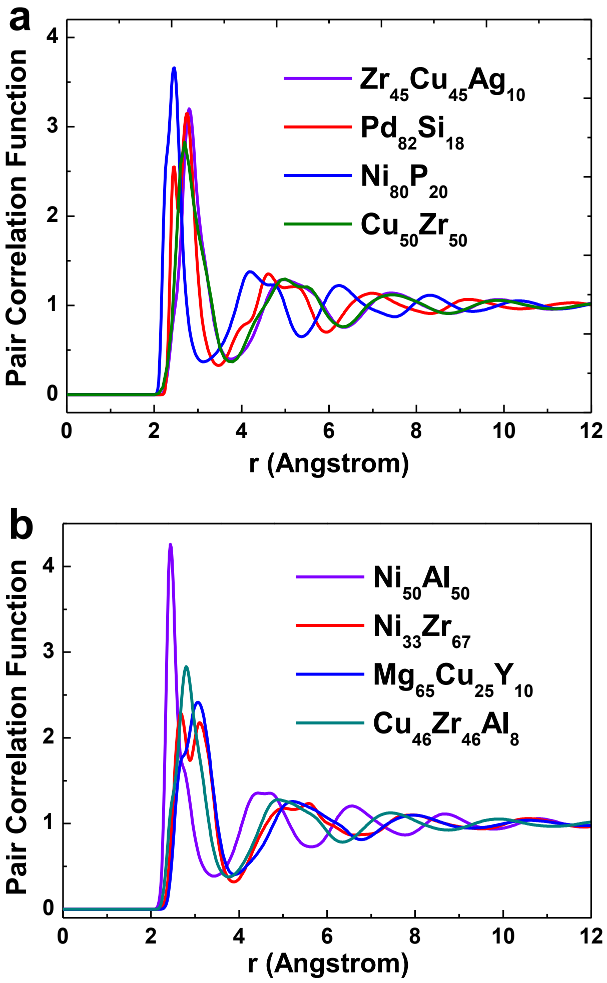

Supplementary Figure 2. Pair correlation functions for different systems at room temperature (300 K) ((**a**) & (**b**)) calculated according to $g(r) = \frac{1}{N\rho}\sum_{i=1}^{N}\sum_{j\neq i}^{N}\langle\delta(\mathbf{r} + \mathbf{r}_j - \mathbf{r}_i)\rangle$. N is the atom number and ρ is the average number density of the system, representing the probability that two atoms are separated by a position vector **r**. The positions of the characteristic peaks are different whereas there occurs a split in the second peak, indicating glassy states in all systems.

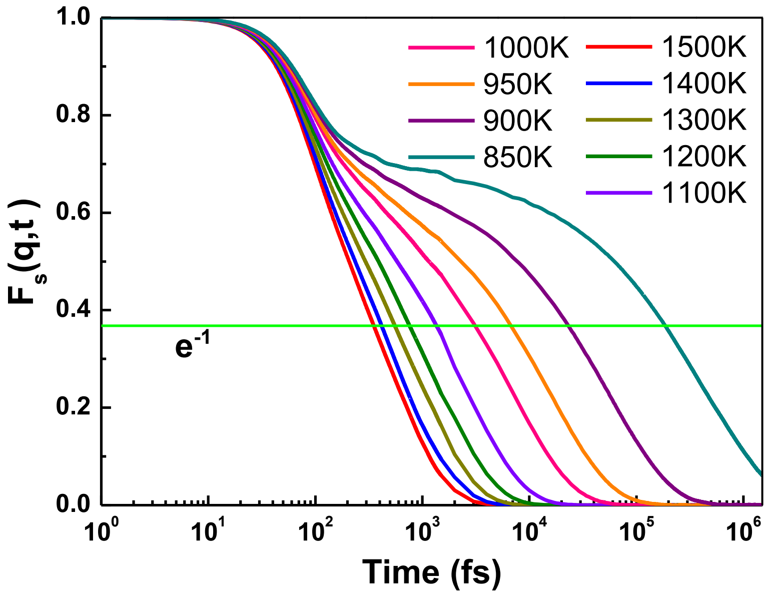

Supplementary Figure 3. Self-intermediate scattering functions $F_s(q,t)$ of $Cu_{46}Zr_{46}Al_8$ metallic glass-forming liquids at various temperatures, from which the α-relaxation time at different temperatures can be extracted.

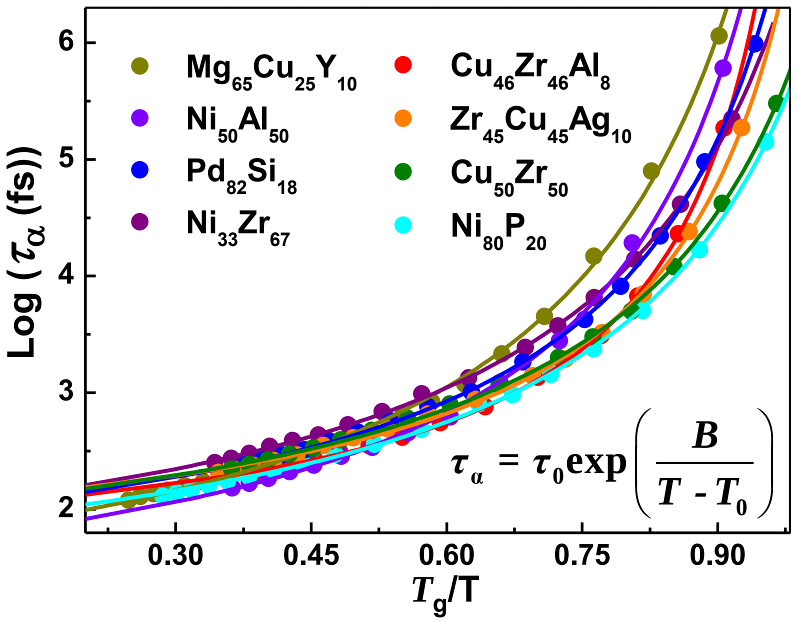

Supplementary Figure 4. The T_g/T dependence of α-relaxation time (τ_α) of the investigated metallic glass-forming liquids. The dotted and solid lines are simulation data and the VFT fittings, respectively.

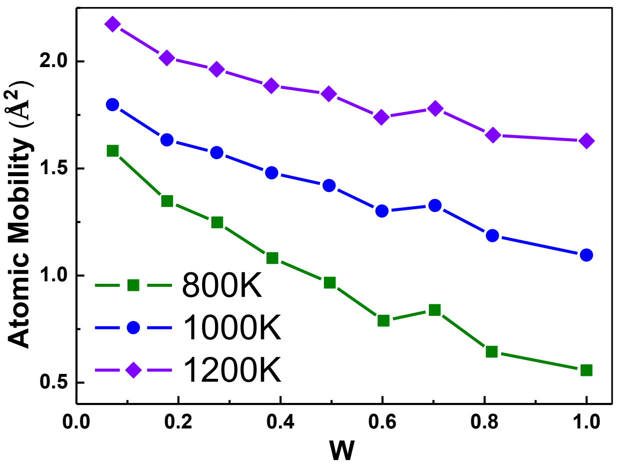

Supplementary Figure 5. Structural basis of atomic mobility in a model system $Cu_{46}Zr_{46}Al_8$ at 800 K, 1000K and 1200 K showing local structure with high five-fold symmetry turns to be slower.

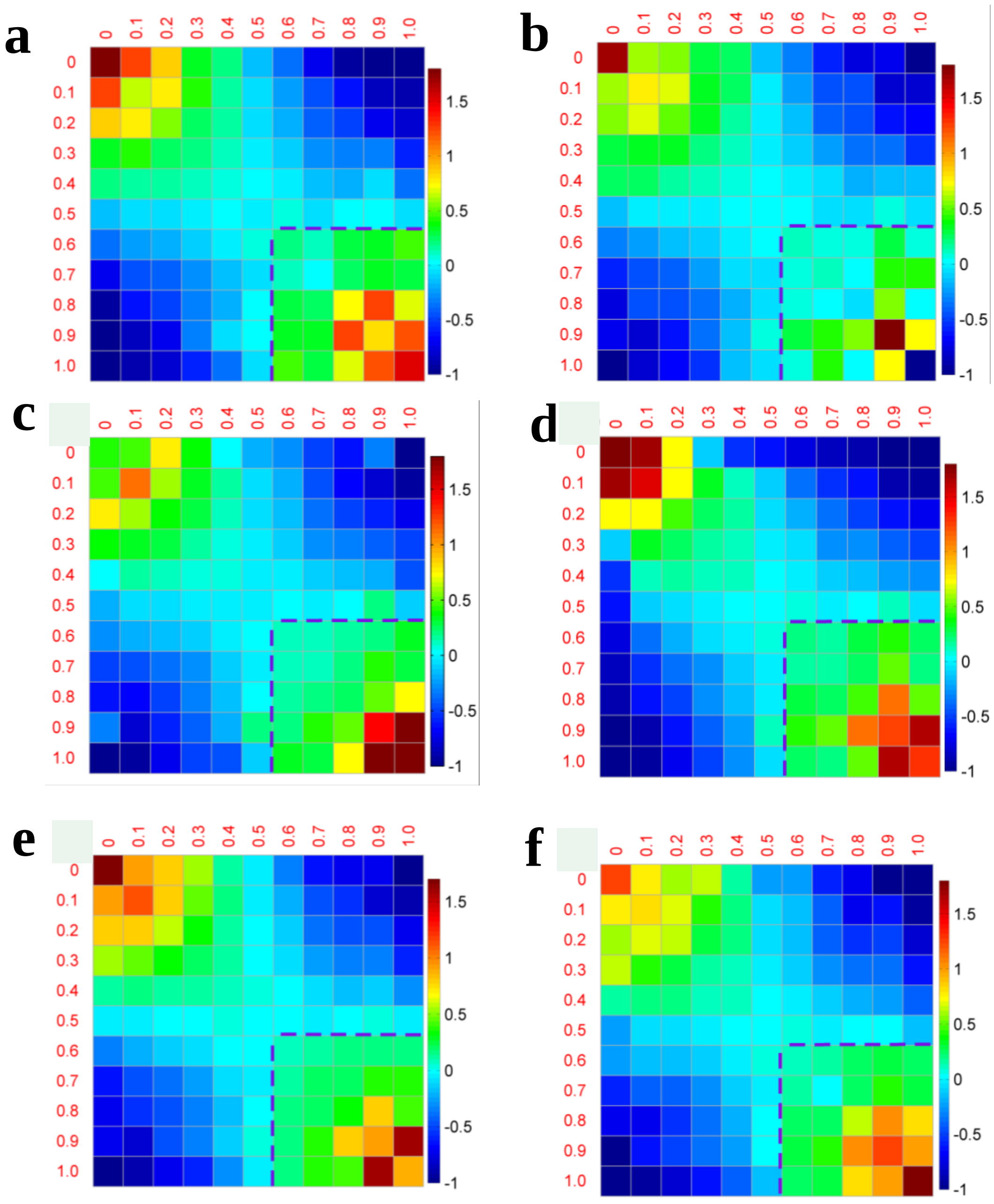

Supplementary Figures 6. The matrices of spatial correlation index C_{ij} of atoms with different degree of five-fold local symmetry at T=1.2T_g for $Cu_{50}Zr_{50}$ (**a**), $Pd_{82}Si_{18}$ (**b**), $Ni_{33}Zr_{67}$ (**c**), $Ni_{50}Al_{50}$ (**d**), $Ni_{80}P_{20}$ (**e**), $Zr_{45}Cu_{45}Ag_{10}$ (**f**), respectively.

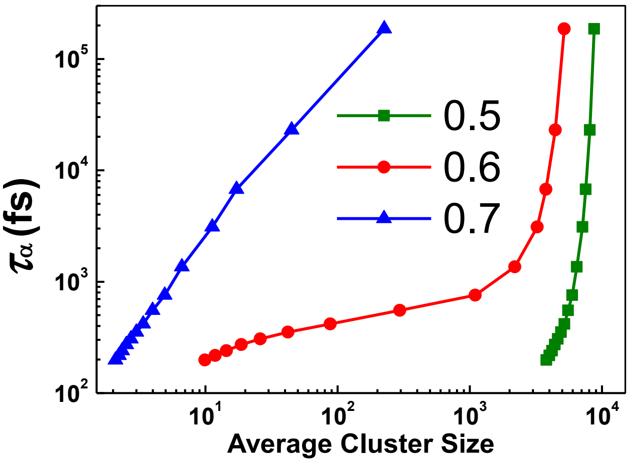

Supplementary Figure 7. Log-log plot of α-relaxation time as a function of the average cluster size with different thresholds of 0.5, 0.6 and 0.7 in $Cu_{46}Zr_{46}Al_8$.

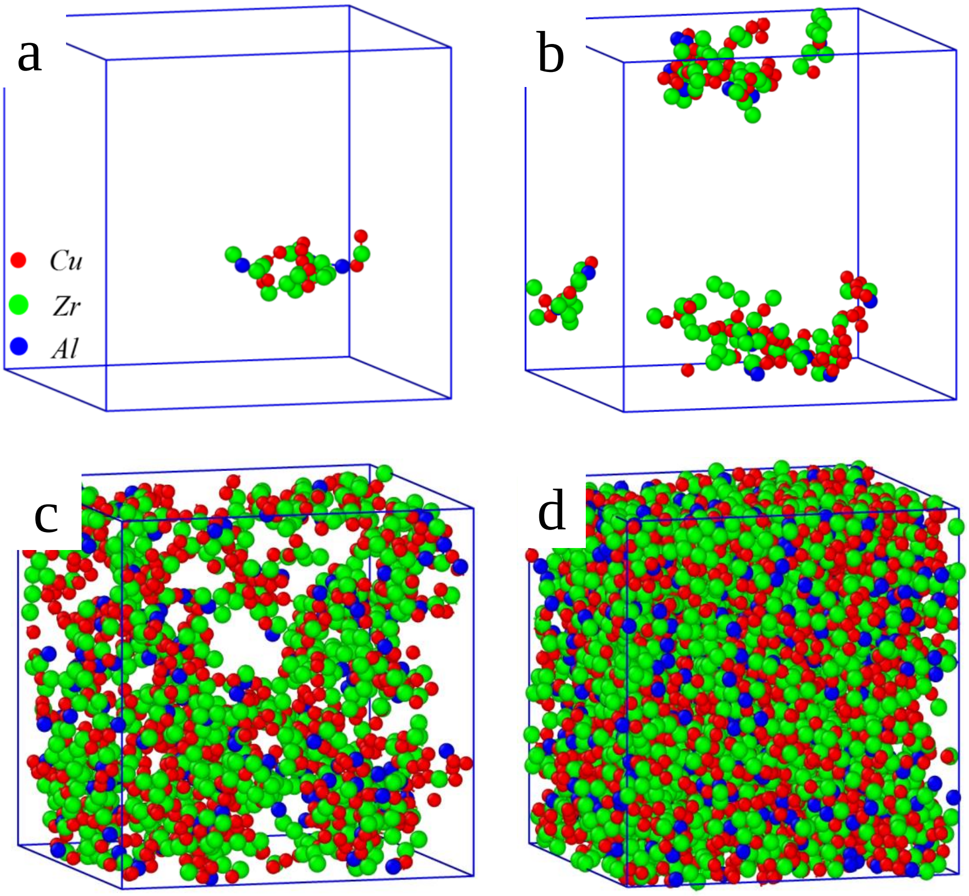

Supplementary Figure 8. Snapshots of the biggest cluster formed by atoms with $f^5 \geq 0.6$ at 2.5T_g(**a**), 2.0T_g(**b**), 1.5T_g(**c**) and 0.9T_g(**d**), respectively (T_g=771 K) in $Cu_{46}Zr_{46}Al_8$ system. The number of atoms involved in is 32, 190, 1726 and 5684 for (**a**)-(**d**), respectively.

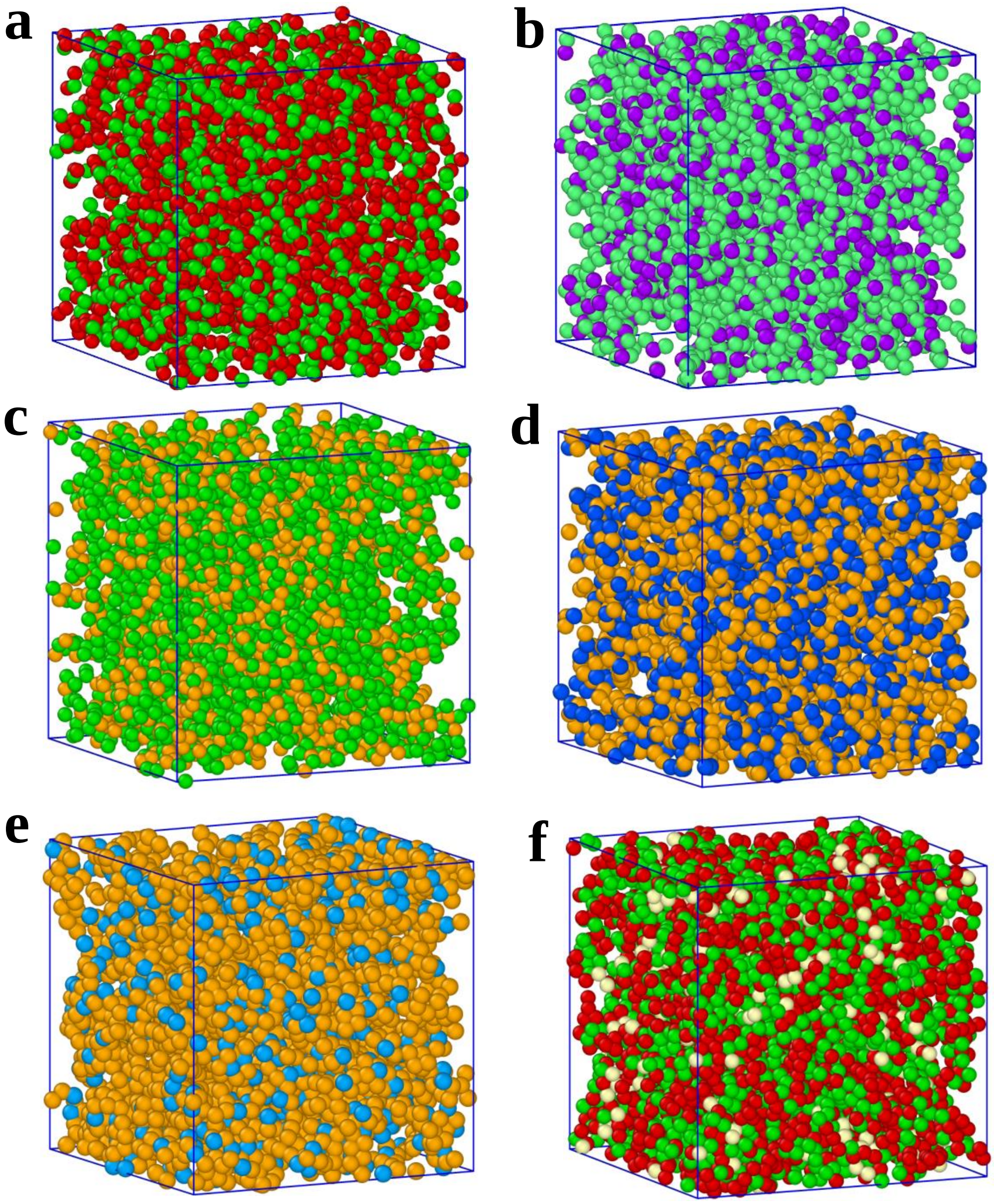

Supplementary Figure 9. The largest cluster formed by atoms with $f^5 \geq 0.6$ at T=0.95T_g for $Cu_{50}Zr_{50}$ (**a**, 3867 atoms), $Pd_{82}Si_{18}$ (**b**, 3457 atoms), $Ni_{33}Zr_{67}$ (**c**,3654 atoms), $Ni_{50}Al_{50}$ (**d**, 3553 atoms), $Ni_{80}P_{20}$ (**e**, 3688 atoms), $Zr_{45}Cu_{45}Ag_{10}$ (**f**, 4823 atoms). The network-like structure forms in each system after glass transition. (dark yellow: Ni, green: Zr, blue: Al, light blue: P, red: Cu, light yellow: Ag, light green: Pd, purple: Si).

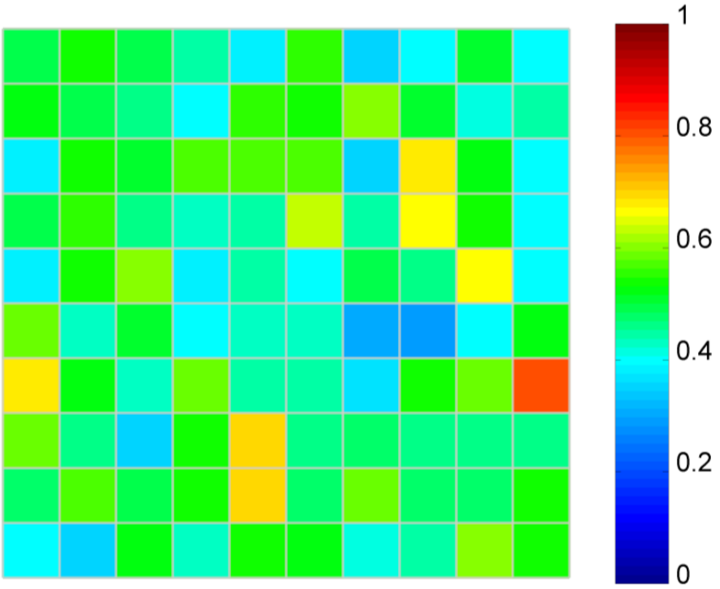

Supplementary Figure 10. Real space distribution of local *W* in a slice with a thickness of 3.3 Å for $Cu_{46}Zr_{46}Al_8$ at 1.2T_g, illustrating the heterogeneous microstructure basis of dynamic heterogeneity according to equation. (5). (box size: 65.88 Å)
